# Geriatric Nutritional Risk Index as a Predictor for Osteoporosis Risk in Elderly Patients with Type 2 Diabetes Mellitus: A Hospital-Based Study

**DOI:** 10.3390/diagnostics16030408

**Published:** 2026-01-27

**Authors:** Abdalla M. Abdelrahman, Michael Edwar Farg, Hanaa A. Nofal, Shaherah Yousef Andargeery, Dina S. Elrafey, Wesam M. R. Ashour, Ahmed Ibrahim Gad

**Affiliations:** 1 Internal Medicine Department, Faculty of Medicine, Zagazig University, Zagazig 44519, Egypt; amirhanin2020@gmail.com (A.M.A.); michaelfarag@medicine.zu.edu.eg (M.E.F.); ahmedgadmed@gmail.com (A.I.G.); 2 Public Health and Community Medicine Department, Faculty of Medicine, Zagazig University, Zagazig 44519, Egypt; dselrafey@gmail.com; 3 Department of Nursing Management and Education, College of Nursing, Princess Nourah bint Abdulrahman University, P.O. Box 84428, Riyadh 11671, Saudi Arabia; 4 Physiology Department, Faculty of Medicine, Zagazig University, Zagazig 44519, Egypt; wesam_ashour@yahoo.com

**Keywords:** GNRI, elderly, osteoporosis, type 2 DM, BMD

## Abstract

**Background**: Osteoporosis is a major complication in older adults with type 2 diabetes mellitus (T2DM). Malnutrition contributes to bone loss, and the Geriatric Nutritional Risk Index (GNRI) has emerged as a simple tool for assessing nutritional status. Evidence on the predictive value of the GNRI for osteoporosis in elderly patients with T2DM remains limited. **Objective**: To evaluate the association between GNRI scores and osteoporosis and determine its predictive performance in elderly patients with T2DM. **Methods**: A cross-sectional study was conducted on 200 elderly patients with T2DM attending the internal medicine outpatient clinics at Zagazig university hospitals between January and October 2025. Clinical data, biochemical parameters, and bone mineral density (BMD) at the lumbar spine, femoral neck, and total hip were assessed. GNRI scores were calculated using standard formulas. Participants were classified into osteoporosis and non-osteoporosis groups according to WHO criteria. Correlations and ROC curve analysis were performed to assess the predictive ability of the GNRI in comparison with age, BMI, and serum albumin. **Results**: Osteoporosis was present in 15% of the cohort. Patients with osteoporosis had significantly lower GNRI scores and lower BMD values at all measured sites (*p* < 0.05). The GNRI showed significant positive correlations with BMD parameters in both sexes. ROC analysis demonstrated that the GNRI had the highest predictive performance for osteoporosis (AUC = 0.80 for all patients; AUC = 0.85 in males; AUC = 0.77 in females). Optimal GNRI cutoff values were <100.03 for the total sample, <99.10 for males, and <100.3 for females. **Conclusions**: The GNRI is a valuable and simple clinical tool for predicting osteoporosis in elderly patients with T2DM. Lower GNRI scores are significantly associated with reduced BMD. Incorporating the GNRI into routine assessment may help identify high-risk patients who require early screening and intervention.

## 1. Introduction

Osteoporosis is a highly prevalent metabolic bone disease characterized by reduced bone mineral density (BMD) and deterioration of bone microarchitecture, resulting in increased bone fragility and susceptibility to fractures [1,2]. It represents a major global public health problem, affecting nearly 200 million individuals worldwide and accounting for approximately 8.9 million fragility fractures annually [3,4]. Osteoporotic fractures are associated with substantial morbidity, mortality, and healthcare costs, particularly among older adults, with one in three women and one in five men over the age of 50 experiencing an osteoporotic fracture during their lifetime [5]. In Europe, osteoporosis-related fragility fractures rank among the leading chronic comorbidities and impose an annual economic burden exceeding €37 billion [6,7].

Osteoporosis affects both sexes, although its epidemiology and pathophysiology differ between men and women. In women, postmenopausal estrogen deficiency is a principal driver of accelerated bone loss, whereas in men, osteoporosis is often underdiagnosed and attributed to age-related hormonal decline, nutritional deficiencies, and secondary causes [8]. Aging itself is associated with progressive bone loss, sarcopenia, and nutritional deterioration, all of which contribute to increased fracture risk irrespective of sex [9].

Type 2 diabetes mellitus (T2DM) is a common metabolic disorder in older adults and is increasingly recognized as an important secondary cause of osteoporosis and fragility fractures. Although individuals with T2DM often present with normal or even increased BMD, epidemiological studies consistently demonstrate a significantly elevated fracture risk, indicating compromised bone quality rather than bone quantity alone [8,10]. The mechanisms linking T2DM to skeletal fragility are multifactorial and include chronic hyperglycemia, oxidative stress, accumulation of advanced glycation end-products in bone collagen, impaired osteoblast function, increased osteoclast activity, microvascular damage, and altered bone remodeling dynamics [11]. These diabetes-related alterations result in structurally weaker bone that is inadequately captured by conventional BMD measurements.

In addition to its direct effects on bone metabolism, T2DM disrupts calcium, phosphorus, and magnesium homeostasis and contributes to systemic inflammation and sarcopenia, further amplifying fracture risk [11]. Clinical data indicate that osteoporosis is highly prevalent among older individuals with T2DM, with reported prevalence rates exceeding 40% in diabetic populations over the age of 50 years, particularly among women [12]. Despite this substantial burden, osteoporosis remains an underrecognized and under-screened complication of T2DM.

Nutritional status has emerged as a critical and potentially modifiable determinant of bone health in elderly individuals, particularly those with chronic metabolic diseases such as T2DM. Malnutrition and protein-energy deficiency negatively affect bone remodeling through reduced osteoblast activity, muscle wasting, impaired balance, and chronic low-grade inflammation, thereby increasing susceptibility to osteoporosis and fractures. The Geriatric Nutritional Risk Index (GNRI), which integrates serum albumin levels and body weight parameters, provides a simple, objective, and clinically feasible assessment of nutritional risk in older adults.

Although the GNRI has been widely studied in relation to mortality, frailty, cardiovascular outcomes, and general prognosis in elderly populations, its role in osteoporosis, particularly as a diabetes-related skeletal complication, remains insufficiently explored [13]. There is a paucity of data specifically evaluating the association between GNRI and osteoporosis in elderly patients with T2DM, and the predictive utility of GNRI for identifying osteoporosis risk in this high-risk population has not been clearly established.

Therefore, the novelty of the present study lies in investigating the GNRI as a nutritional-based predictive marker for osteoporosis specifically in elderly patients with type 2 diabetes mellitus, addressing an important gap in current diabetes-related bone disease research. The aim of this study was to evaluate the association between GNRI and osteoporosis and to assess its predictive performance in elderly individuals with T2DM.

## 2. Patients and Methods

### 2.1. Study Design

This was a cross-sectional analytical study conducted between January 2025 and October 2025 at the Internal Medicine Outpatient Clinics of Zagazig University Hospitals, Egypt. The study was conducted in accordance with the Declaration of Helsinki. Ethical approval was obtained from the Zagazig University Institutional Review Board (ZU-IRB#1048/29 January 2025), and written informed consent was obtained from all participants prior to enrollment.

### 2.2. Sample Size

Sample size calculation was performed using MedCalc Statistical Software (MedCalc Software Ltd., Ostend, Belgium) for ROC curve analysis. With an AUC of 0.70 and an osteoporosis prevalence of 15.1% [13], a two-sided alpha level of 0.05, 80% power, and considering the finite population size (N = 450), the minimum required sample size was 150 participants.

### 2.3. Study Population

The study was conducted on 200 patients (114 males and 86 females), with mean age 64.39 ± 8.3 years. Inclusion criteria included patients with type 2 diabetes mellitus who had a diagnosis based on American Diabetes Association criteria, age ≥ 60 years of both sexes, and the study excluded any patients with malignancy, severe cardiac, hepatic, or renal disease, endocrine disorders affecting bone (thyroid, parathyroid, pituitary, adrenal, or gonadal diseases), long-term bedridden status, chronic corticosteroid therapy, active metabolic bone diseases other than osteoporosis, or current use of medications affecting bone metabolism (e.g., calcium supplements, vitamin D, bisphosphonates, denosumab) that were initiated within the previous 6 months, to minimize confounding effects on bone metabolism while limiting selection bias.

To allow meaningful comparison, participants were classified into an osteoporosis group (T-score ≤ −2.5) and a non-osteoporosis control group (T-score > −2.5, including normal bone density and osteopenia).

**Figure 1:**
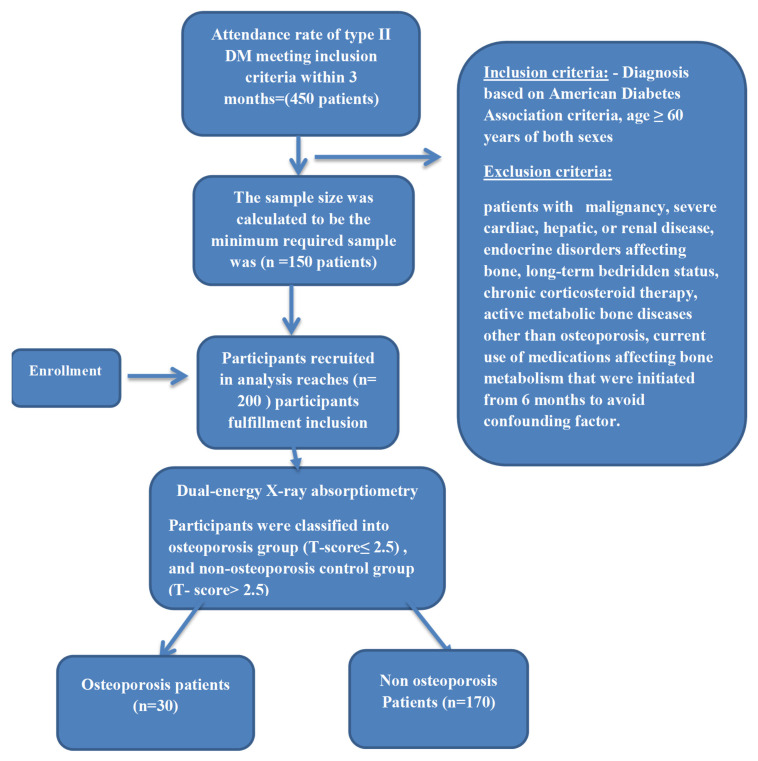
Flowchart of studied participants.

A study flowchart illustrating patient screening, eligibility assessment, exclusions, and final group allocation is provided in Figure 1 to enhance clarity of the study design.

### 2.4. Data Collection

Patients’ demographics and clinical characteristics, including information on age, sex, and comorbidities, were collected from their respective medical records. The duration of diabetes was counted by years, starting from the time point the patient was diagnosed with type 2 diabetes mellitus based on the medical record, up to carrying out the blood tests and measuring bone mineral density (BMD) values. Body weight and height were measured with light clothing and without shoes. The body mass index (BMI; kg/m^2^), defined as the body weight (kg) per square of height (m^2^), was calculated for each patient. Blood pressure (mmHg) was measured by a mercury sphygmomanometer in the supine position after resting for 5 min. Smoking history was recorded (smoking status was categorized as never smoker, former smoker (ceased ≥12 months prior), or current smoker). Alcohol intake was recorded as none or current use, with frequency documented when applicable.

## 3. Biochemical Indicators

Fasting venous blood samples were collected at 6:00 a.m. after an overnight fast of at least 8 h. Fasting blood glucose (mmol/L) and glycated hemoglobin (HbA1c) (mmol/L) were measured using standardized enzymatic methods in the central laboratory. Serum lipid profile (total cholesterol (mmol/L), triglycerides (mmol/L), HDL-C (mmol/L), LDL-C (mmol/L), albumin (g/L), creatinine (µmol/L), albumin-corrected calcium, and 25-hydroxyvitamin D levels (ng/mL) were measured using automated analyzers according to manufacturer protocols.

All biochemical measurements were performed following standardized laboratory procedures as previously described in international clinical chemistry guidelines.

### 3.1. Bone Mineral Density (BMD Index)

Dual-energy X-ray absorptiometry (Hologic-Discovery, Bedford, MA, USA) was used to measure the BMD value of each patient at three positions: total lumbar spine (L1–4), femur neck, and total hip. In accordance with the criteria for the definition of osteoporosis in the 1994 WHO report, T values ≤ −2.5 standard deviations were obtained for any part of the lumbar spine, femoral neck, or total hip [14].

### 3.2. Geriatric Nutritional Risk Index (GNRI)

The GNRI was calculated using the following formula by Bouillanne et al. [15]: GNRI = (1.489 × serum albumin [g/L]) + (41.7 × actual body weight/ideal body weight).

Serum albumin was consistently measured and expressed in g/L. Ideal body weight was calculated as follows: Men: IBW = height (cm) − 100 − [(height − 150)/4], Women: IBW = height (cm) − 100 − [(height − 150)/2].

### 3.3. Statistical Analysis

The collected data were computerized and statistically analyzed using SPSS program (Statistical Package for Social Science) version 27.0 (IBM, 2020). Qualitative data were represented as frequencies and relative percentages. The Chi square test was used to calculate difference between qualitative variables. Quantitative data were tested for normality using the KS (Kolmogorov–Smirnov) test and expressed as mean ± SD (standard deviation) or median and range. The independent *t* test and Mann–Whitney test were used to calculate difference between quantitative variables. Pearson’s correlation coefficient was used to calculate correlation between quantitative variables. Receiver operating characteristic (ROC) curve analysis was used to identify optimal cutoff values of different parameters with maximum sensitivity and specificity for prediction of the osteoporosis. Reliability data were calculated using Sensitivity, Specificity, PV+, PV−, and Accuracy (*p* value of <0.05 indicates significant results; *p* value of <0.001 indicates highly significant results).

## 4. Results

Table 1 presents the demographic and clinical characteristics of the studied 200 elderly patients with type II DM. The mean age of the studied patients was 64.39 ± 8.3 years, and the mean BMI was 24.66 ± 3.1 kg/m^2^. Regarding sex, 57% of patients were males and 43% of them were females. There was no statistically significant difference between males and females regarding diabetes duration, DBP, Ca, LDL, and GNRI score. Age and BMI were significantly higher in females than males (*p* = 0.02 and 0.009, respectively), while smoking and drinking alcohol were significantly higher in males than females (*p* < 0.001 in both). Systolic blood pressure was significantly higher in females than males (*p* = 0.04). There was statistically significant difference between males and females regarding FBG, HbA1c, TC, TG, HDL, albumin, and creatinine. There was no statistically significant difference between males and females regarding GNRI scores. Serum 25(OH)D and BMD was notably significantly lower in females, leading to a higher prevalence of osteoporosis (OR = 2.66 CI 95% 1.19–5.93).

Table 2 shows that BMD at total lumbar, femur neck, and total hip were all significantly lower in cases with osteoporosis compared to cases without osteoporosis (*p* = 0.04, <0.001, <0.001, respectively). GNRI scores were also significantly lower in cases with osteoporosis compared to cases without osteoporosis (*p* < 0.001 and effect size d = 1 CI 95% 0.68–1.49).

Table 3 demonstrates the correlation between the GNRI and different parameters. There were significant positive correlations between the GNRI and various bone mineral densities (BMDs) in both males and females, indicating that higher GNRI scores are associated with better bone health and significant positive correlation with albumin-corrected calcium, while there was no significant correlation between the GNRI and serum 25(OH)D levels.

**Figure 2:**
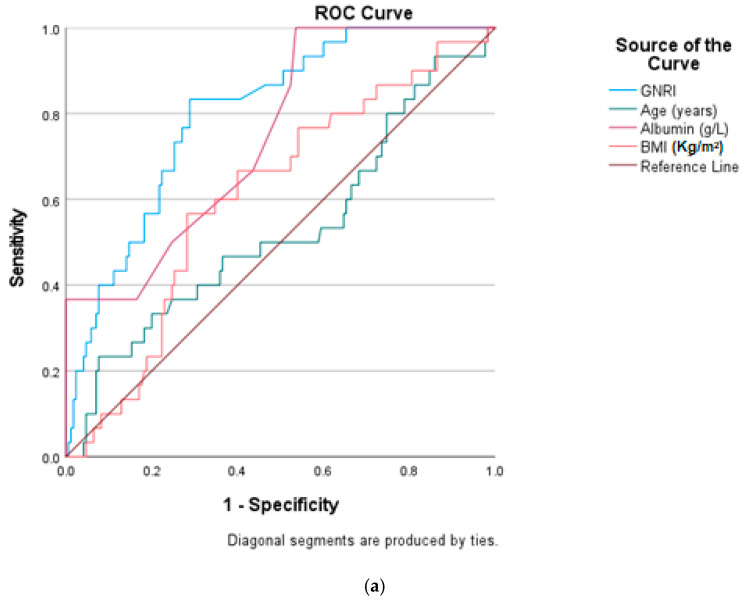

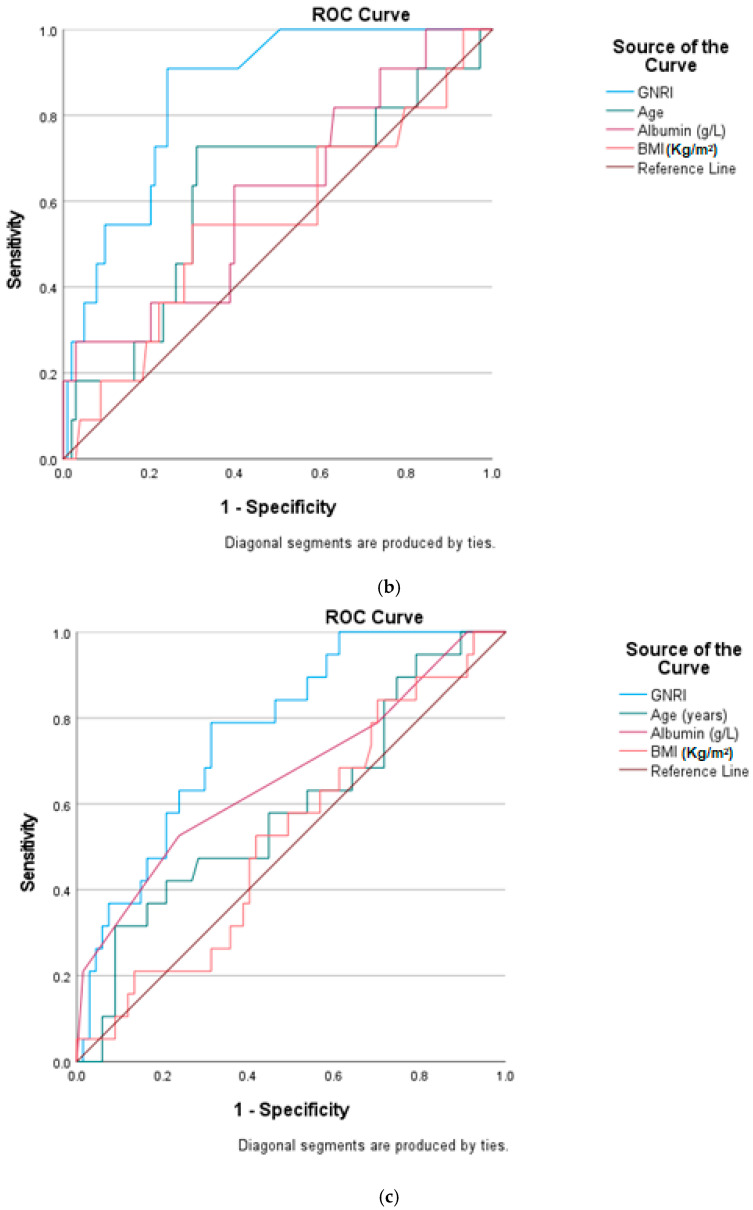
(**a**–**c**): Predictive properties of the GNRI, age, BMI, and serum albumin for osteoporosis among all the studied patients, male, and female patients.

Table 4 and Figure 2a–c show that the predictabilities of the GNRI, serum albumin, BMI, and age for osteoporosis were estimated by the ROC curves. Compared with serum albumin, BMI, and age, the AUC of the GNRI was the highest and statistically significant (*p* < 0.001), (0.85 for men and 0.77 for women and 0.80 for all). The optimal cutoff of the GNRI for predicting osteoporosis was <100.03 with a sensitivity of 83.3% and a specificity of 71.8% in all; <99.10 with a sensitivity of 90.9% and a specificity of 75.7% in males; and <100.3 with a sensitivity of 78.9% and a specificity of 68.7% in females.

## 5. Discussion

In this hospital-based cross-sectional study of 200 elderly patients with type 2 diabetes mellitus (T2DM), the Geriatric Nutritional Risk Index (GNRI) demonstrated a strong and consistent association with osteoporosis. Patients with osteoporosis had significantly lower GNRI scores, and the GNRI showed significant positive correlations with bone mineral density (BMD) parameters at the lumbar spine, femoral neck, and total hip. Receiver operating characteristic (ROC) analysis further demonstrated that the GNRI outperformed age, body mass index (BMI), and serum albumin alone in predicting osteoporosis, supporting its potential role as an integrated nutritional risk indicator in this high-risk population.

The association between the GNRI and skeletal health is biologically plausible and consistent with the existing literature highlighting malnutrition as a key contributor to osteoporosis in older adults. Protein-energy malnutrition adversely affects osteoblast activity, impairs collagen synthesis, and reduces the availability of essential micronutrients, including calcium and vitamin D. Moreover, malnutrition promotes chronic low-grade inflammation and muscle wasting, both of which accelerate bone loss and increase fracture risk. Previous studies conducted in community-dwelling elderly individuals, hospitalized geriatric populations, and patients with chronic diseases such as chronic kidney disease, chronic obstructive pulmonary disease, and cardiovascular disease have reported similar associations between low GNRI scores and reduced BMD or osteoporosis [16,17,18,19,20,21].

The present study extends these findings to elderly individuals with T2DM, a population at higher risk of compromised bone quality due to chronic hyperglycemia, oxidative stress, accumulation of advanced glycation end-products, and microarchitectural deterioration despite frequently normal or even elevated BMD values [15,22,23].

The GNRI incorporates serum albumin and the ratio of actual to ideal body weight, both of which serve as markers of protein-energy status, muscle reserve, and overall nutritional adequacy. Albumin, although influenced by inflammation and hydration, remains a widely used indicator of nutritional risk in older adults [24].

Low body weight or relative weight deficiency may reflect reduced muscle mass and functional reserve. While sarcopenia and sarcopenic obesity were not directly assessed in the present study, the observed superiority of the GNRI over BMI suggests that combined nutritional indices may better reflect underlying alterations in body composition than weight-based measures alone [25,26]. BMI is unable to differentiate between fat and lean mass and may therefore underestimate skeletal risk in elderly patients with T2DM, in whom sarcopenic obesity is common and excess adiposity may mask significant muscle loss [27]. This limitation should be acknowledged when interpreting the role of body composition in the observed associations.

An interesting finding of this study is the higher predictive performance of the GNRI in males compared with females. This may be attributed to greater variability in muscle mass and nutritional reserves among men, allowing the GNRI to better distinguish osteoporotic from non-osteoporotic individuals. In elderly women, bone loss is more strongly driven by postmenopausal hormonal changes, which may lessen the relative impact of nutritional factors. Sex differences in fat distribution, inflammation, physical activity, and comorbidity burden, as well as the under recognition of secondary osteoporosis in men, potentially amplifying the predictive value of the GNRI in male patients [25,28].

The optimal GNRI cutoff values identified in this study (approximately 99–100) are consistent with previously reported thresholds for adverse clinical outcomes in geriatric populations. Values below 100 have been associated with increased morbidity, frailty, hospitalization, disability, and mortality [24,29]. The current findings reinforce the utility of this cutoff in predicting skeletal fragility as well.

From an ontogenetic perspective, osteoporosis and T2DM are age-related conditions that share common mechanisms such as chronic inflammation, hormonal changes, and loss of muscle and bone mass. Aging amplifies the adverse effects of diabetes on bone remodeling, making nutritional status increasingly important in late life. The GNRI may therefore reflect the cumulative metabolic and nutritional burden contributing to bone deterioration over the life course. Serum albumin alone performed moderately but remained inferior to the GNRI, emphasizing the advantage of combining biochemical and anthropometric parameters.

These findings have meaningful clinical implications. The GNRI is a simple, inexpensive, and easily applicable index that can be incorporated into routine outpatient care. Given that DXA screening is not universally accessible, particularly in low-resource settings, the GNRI may serve as a preliminary risk stratification tool to identify elderly diabetic patients who may benefit from further bone health assessment.

Early identification of high-risk individuals may support timely interventions, including nutritional optimization, vitamin D and calcium repletion, resistance exercise programs, and prioritization for DXA evaluation rather than replacement of standard diagnostic methods.

However, the GNRI is an indirect marker and does not directly measure bone mineral density. Its interpretation may be influenced by factors such as inflammation, acute illness, or hypoalbuminemia unrelated to nutritional status. Therefore, the GNRI should be viewed as complementary to, rather than a substitute for, DXA-based diagnosis.

Given the overlap between malnutrition, sarcopenia, and osteoporosis, improving nutritional status may play a supportive preventive role in reducing fracture risk and enhancing physical function, while definitive diagnosis and management decisions should continue to rely on established imaging

This study has limitations. Its cross-sectional design precludes causal inference between nutritional status and osteoporosis. Longitudinal studies are needed to evaluate whether the GNRI predicts future BMD decline or incident fractures. The hospital-based sample may limit generalizability to community-based older adults. Additionally, bone turnover markers (such as P1NP and CTX) and inflammatory markers (such as CRP and IL-6) were not assessed, which could have provided mechanistic insights. Despite these limitations, the relatively large sample size and standardized BMD assessment strengthen the validity of the results.

Overall, the findings highlight the importance of integrating nutritional evaluation into routine diabetes care for elderly individuals. The GNRI emerged as a reliable and practical tool that surpassed traditional parameters in predicting osteoporosis. Incorporating the GNRI into clinical pathways may facilitate early detection of nutritionally vulnerable patients and support targeted preventive strategies aimed at improving bone health and reducing fracture risk.

## 6. Conclusions

The GNRI is a robust and practical predictor of osteoporosis in elderly patients with T2DM. Lower GNRI levels are associated with reduced BMD and a higher likelihood of osteoporosis. The GNRI outperformed age, BMI, and serum albumin in predictive accuracy. Routine use of the GNRI may facilitate early identification of high-risk individuals and support the implementation of timely nutritional and osteoporosis-focused interventions.

## Figures and Tables

**Table 1:** Basic characteristics of the studied patients.

Variable	Total (*n* = 200)	Male (*n* = 114)	Female (*n* = 86)	Test	*p*
Age: (years)	64.39 ± 8.3	63.19 ± 8.57	65.98 ± 7.69	2.39 ^#^	0.02 *
Smoking:	57 (28.5%)	56 (49.1%)	1 (1.2%)	55.33 ^^^	<0.001 **
Drinking:	43 (21.5%)	42 (36.8%)	1 (1.2%)	36.97 ^^^	<0.001 **
BMI (Kg/m^2^)	24.66 ± 3.1	24.17 ± 2.65	25.82 ± 3.53	2.63 ^#^	0.009 *
Diabetes duration (years)	7.74 (0.64–30)	7.03 (0.64–30)	8.38 (1–22.75)	1.44 ^$^	0.15 NS
Systolic blood pressure (mmHg)	137.9 ± 19.82	135.41 ± 18.55	141.2 ± 21.04	2.06 ^#^	0.04 *
Diastolic blood pressure (mmHg)	77.27 ± 11.26	77.58 ± 12.14	76.87 ± 10.05	0.44 ^#^	0.66 NS
FBG (mmol/L)	9.08 ± 2.45	8.64 ± 2.43	9.66 ± 2.36	3.00 ^#^	0.003 *
HbA1c (mmol/L)	9.89 ± 2.09	10.44 ± 1.99	9.16 ± 2.01	4.48 ^#^	<0.001 **
TC (mmol/L)	4.51 ± 1.36	4.18 ± 1.25	4.94 ± 1.38	4.05 ^#^	<0.001 **
TG (mmol/L)	1.94 ± 0.91	1.63 ± 0.68	2.35 ± 1.02	6.02 ^#^	<0.001 **
HDL (mmol/L	1.09 ± 0.32	0.92 ± 0.28	1.32 ± 0.21	11.35 ^#^	<0.001 **
LDL (mmol/L)	2.80 ± 0.92	2.73 ± 0.92	2.9 ± 0.9	1.32 ^#^	0.19 NS
Albumin (g/L)	36.77 ± 4.03	37.37 ± 3.83	35.98 ± 4.18	2.45 ^#^	0.02 *
Creatinine (µmol/L)	72.41 ± 25.26	77.74 ± 24.73	65.35 ± 24.34	3.53 ^#^	<0.001 **
25(OH)D (ng/mL)	59.15 ± 17.02	61.54 ± 17.59	55.99 ± 15.79	2.31 ^#^	0.02 *
Calcium (mmol/L)	2.48 ± 0.22	2.46 ± 0.23	2.51 ± 0.19	1.31 ^#^	0.19 NS
Total lumbar (g/cm^2^)	1.03 ± 0.35	1.09 ± 0.34	0.96 ± 0.35	2.81 ^#^	0.005 *
Femur neck (g/cm^2^)	0.81 ± 0.16	0.85 ± 0.21	0.75 ± 0.01	4.37 ^#^	<0.001 **
Total hip (g/cm^2^)	0.85 ± 0.11	0.90 ± 0.09	0.79 ± 0.10	7.59 ^#^	<0.001 **
Osteoporosis:	30 (15%)	11 (9.6%)	19 (22.1%)	5.95 ^^^	0.02 *
GNRI	101.91 ± 7.52	101.76 ± 6.75	102.1 ± 8.46	0.31 ^#^	0.75 NS

Data presented as mean ± SD, median (range), or N (%); ^#^: Independent *t* test; ^$^: Mann–Whitney test; ^^^: Chi square test (χ^2^); NS: Non-significant (*p* > 0.05); *: Significant (*p* < 0.05); **: Highly significant (*p* < 0.001); FBG: fasting blood glucose; TC: total cholesterol; TG: triglyceride; HDL: high-density lipoprotein; LDL: low-density lipoprotein; 25(OH)D: 25-hydroxy-vitamin D; GNRI: Geriatric Nutritional Risk Index; BMD: bone mineral density.

**Table 2:** Basic characteristics of the studied patients according to osteoporosis.

Variable	No Osteoporosis (*n* = 170)	Osteoporosis (*n* = 30)	Test	*p*
Age: (years)	64.28 ± 8.25	65.04 ± 8.68	0.46 ^#^	0.64 NS
Smoking:	52 (30.6%)	5 (16.7%)	2.43 ^^^	0.12 NS
Drinking:	37 (21.8%)	6 (20%)	0.05 ^^^	0.83 NS
BMI (Kg/m^2^)	24.61 ± 3.10	24.97 ± 3.19	0.59 ^#^	0.55 NS
Diabetes duration (years)	7.86 (0.75–22.75)	7.08 (0.64–30)	0.39 ^$^	0.70 NS
Systolic blood pressure (mmHg)	137.78 ± 20.51	138.58 ± 15.60	0.20 ^#^	0.48 NS
Diastolic blood pressure (mmHg)	77.84 ± 11.4	74.05 ± 10.01	1.71 ^#^	0.09 NS
FBG (mmol/L)	9.08 ± 2.45	9.07 ± 2.48	0.01 ^#^	0.99 NS
HbA1c (mmol/L)	9.97 ± 2.09	9.43 ± 2.08	1.31 ^#^	0.19 NS
TC (mmol/L)	4.46 ± 1.32	4.8 ± 1.57	1.27 ^#^	0.21 NS
TG (mmol/L)	1.95 ± 0.94	1.86 ± 0.76	0.51 ^#^	0.61 NS
HDL (mmol/L	1.08 ± 0.33	1.13 ± 0.31	0.85 ^#^	0.40 NS
LDL (mmol/L)	2.81 ± 0.9	2.76 ± 1.00	0.27 ^#^	0.79 NS
Albumin (g/L)	36.78 ± 4.02	36.74 ± 4.16	0.05 ^#^	0.96 NS
Creatinine (µmol/L)	72.32 ± 25.49	72.93 ± 24.35	0.12 ^#^	0.90 NS
25(OH)D (ng/mL)	59.17 ± 17.32	59.06 ± 15.49	0.03 ^#^	0.97 NS
Calcium (mmol/L)	2.47 ± 0.22	2.51 ± 0.20	0.86 ^#^	0.39 NS
Total lumbar (g/cm^2^)	1.04 ± 0.37	0.90 ± 0.024	1.99 ^#^	0.04 *
Femur neck (g/cm^2^)	0.83 ± 0.15	0.65 ± 0.15	6.08 ^#^	<0.001 **
Total hip (g/cm^2^)	0.87 ± 0.09	0.74 ± 0.12	6.90 ^#^	<0.001 **
GNRI	103.05 ± 7.20	95.42 ± 5.84	5.50 ^#^	<0.001 **

Data presented as mean ± SD, median (range), or N (%); ^#^: Independent *t* test; ^$^: Mann–Whitney test; ^^^: Chi square test (χ^2^); NS: Non-significant (*p* > 0.05); *: Significant (*p* < 0.05); **: Highly significant (*p* < 0.001); FBG: fasting blood glucose; TC: total cholesterol; TG: triglyceride; HDL: high-density lipoprotein; LDL: low-density lipoprotein; 25(OH)D: 25-hydroxy-vitamin D; GNRI: Geriatric Nutritional Risk Index; BMD: bone mineral density.

**Table 3:** Correlation between GNRI and different parameters among the studied patients.

Variable	GNRI					
	Total (*n* = 200)		Male (*n* = 114)		Female (*n* = 86)	
	r	*p*	r	*p*	r	*p*
Age: (years)	−0.07	0.35 NS	−0.09	0.33 NS	−0.05	0.64 NS
BMI (Kg/m^2^)	−0.03	0.69 NS	0.05	0.58 NS	−0.10	0.36 NS
D.M duration (years)	0.11	0.14 NS	0.13	0.17 NS	0.08	0.49 NS
SBP (mmHg)	0.02	0.83 NS	−0.08	0.42 NS	0.09	0.39 NS
DBP (mmHg)	0.07	0.32 NS	0.03	0.77 NS	0.13	0.23 NS
FBG (mmol/L)	0.09	0.19 NS	0.05	0.60 NS	0.14	0.21 NS
HbA1c (mmol/L)	−0.01	0.84 NS	−0.03	0.79 NS	0.01	0.93 NS
TC (mmol/L)	−0.11	0.13 NS	−0.12	0.22 NS	−0.12	0.27 NS
TG (mmol/L)	−0.11	0.12 NS	−0.07	0.74 NS	−0.18	0.11 NS
HDL (mmol/L	−0.13	0.08 NS	0.18	0.06 NS	−0.17	0.10 NS
LDL (mmol/L)	−0.03	0.65 NS	−0.04	0.69 NS	−0.03	0.76 NS
Albumin (g/L)	−0.11	0.11 NS	−0.001	0.99 NS	−0.06	0.56 NS
Creatinine (µmol/L)	0.01	0.90 NS	0.02	0.85 NS	0.01	0.92 NS
25(OH)D (ng/mL)	0.08	0.26 NS	0.16	0.10 NS	0.004	0.97 NS
Calcium (mmol/L)	0.22	0.03 *	0.31	0.008	0.28	0.01 *
Total lumbar (g/cm^2^)	0.21	0.04 *	0.22	0.03 *	0.27	0.02 *
Femur neck (g/cm^2^)	0.34	<0.001 **	0.30	0.009 *	0.36	<0.001 **
Total hip (g/cm^2^)	0.29	0.01 *	0.25	0.02 *	0.30	0.009 *

r: Pearson’s correlation coefficient; NS: Non-significant (*p* > 0.05); *: Significant (*p* < 0.05); **: Highly significant (*p* < 0.001); FBG: fasting blood glucose; TC: total cholesterol; TG: triglyceride; HDL: high-density lipoprotein; LDL: low-density lipoprotein; 25(OH)D: 25-hydroxy-vitamin D.

**Table 4:** Predictive properties of the GNRI, age, BMI, and albumin for osteoporosis among the studied patients.

Parameters		Age	BMI	Albumin	GNIR
Total	AUC	0.53	0.61	0.75	0.80
	95% CI	0.41–0.65	0.51–0.71	0.66–0.84	0.73–0.88
	*p*	0.62 NS	0.054 NS	<0.001 **	<0.001 **
	Cutoff	>72	>25.5	<32.39	<100.03
	Sensitivity	50%	56.7%	78.6%	83.3%
	Specificity	54.7%	62.4%	63.3%	71.8%
	PPV	16.3%	20.9%	27.9%	34.2%
	NPV	86.1%	89%	94.7%	96.1%
	Accuracy	54%	61.5%	66%	73.5%
Male	AUC	0.62	0.55	0.62	0.85
	95% CI	0.44–0.81	0.36–0.75	0.44–0.79	0.76–0.94
	*p*	0.18 NS	0.58 NS	0.21 NS	<0.001 **
	Cutoff	>69	>24.67	<34.2	<99.10
	Sensitivity	72.7%	54.5%	63.3%	90.9%
	Specificity	68.9%	66%	60.2%	75.7%
	PPV	20%	14.6%	14.6%	28.6%
	NPV	95.9%	93.1%	93.9%	98.7%
	Accuracy	69.3%	64.9%	60.5%	77.2%
Female	AUC	0.59	0.53	0.67	0.77
	95% CI	0.44–0.74	0.39–0.67	0.52–0.81	0.99–0.88
	*p*	0.23 NS	0.72 NS	0.03 *	<0.001 **
	Cutoff	>66.63	>26.22	<35	<100.3
	Sensitivity	57.9%	52.6%	73.7%	78.9%
	Specificity	55.2%	58.2%	65.7%	68.7%
	PPV	26.8%	26.3%	37.8%	41.7%
	NPV	82.2%	81.3%	89.8%	92%
	Accuracy	55.8%	57%	67.4%	70.9%

AUC: Area under curve; CI: Confidence interval; PPV: Positive predicted value; NPV: Negative predicted value; NS: Non-significant (*p* > 0.05); *: Significant (*p* < 0.05); **: Highly significant (*p* < 0.001); GNRI: Geriatric Nutritional Risk Index.

## Data Availability

The data sets used and/or analyzed during the current study are available from the corresponding author on reasonable request.

## References

[1] Gkastaris K., Goulis D.G., Potoupnis M., Anastasilakis A.D., Kapetanos G. (2020). Obesity, osteoporosis and bone metabolism. J. Musculoskelet. Neuronal Interact..

[2] Li Z., Liu P., Yuan Y., Liang X., Lei J., Zhu X., Zhang Z., Cai L. (2021). Loss of longitudinal superiority marks the microarchitecture deterioration of osteoporotic cancellous bones. Biomech. Model. Mechanobiol..

[3] Sözen T., Özışık L., Başaran N.Ç. (2017). An overview and management of osteoporosis. Eur. J. Rheumatol..

[4] Rundasa D.T., Ayisa A.A., Mekonen E.G. (2022). Knowledge, Health Belief, and Associated Factors Towards the Prevention of Osteoporosis Among Postmenopausal Women in Debre Birhan City Central Ethiopia. Int. J. Orthop. Trauma Nurs..

[5] Devi S., Sahu S., Subhiksha D., Behera K.K., Priyadarsini N., Dey A., Sahoo D., Dash A. (2025). Prevalence and risk factors of osteoporosis in diabetic individuals above 50 years of age at a tertiary care hospital: An observational study. J. Clin. Transl. Res..

[6] Hernlund E., Svedbom A., Ivergård M., Compston J., Cooper C., Stenmark J., McCloskey E.V., Jönsson B., Kanis J.A. (2013). Osteoporosis in the European Union: Medical management, epidemiology and economic burden. A report prepared in collaboration with the International Osteoporosis Foundation (IOF) and the European Federation of Pharmaceutical Industry Associations (EFPIA). Arch. Osteoporos..

[7] Borgström F., Karlsson L., Ortsäter G., Norton N., Halbout P., Cooper C., Lorentzon M., McCloskey E.V., Harvey N.C., Javaid M.K. (2020). Fragility fractures in Europe: Burden, management and opportunities. Arch. Osteoporos..

[8] Rinonapoli G., Ruggiero C., Meccariello L., Bisaccia M., Ceccarini P., Caraffa A. (2021). Osteoporosis in men: A review of an underestimated bone condition. Int. J. Mol. Sci..

[9] Hatta N.N.K.N.M., Firdaus M.K.Z.H., Hasan M.K.C. (2023). Enhancing Bone Health in Aging Populations to Prevent Fragility Fractures. Int. J. Care Sch..

[10] Eller-Vainicher C., Cairoli E., Grassi G., Grassi F., Catalano A., Merlotti D., Falchetti A., Gaudio A., Chiodini I., Gennari L. (2020). Pathophysiology and management of type 2 diabetes mellitus bone fragility. J. Diabetes Res..

[11] De Martinis M., Allegra A., Sirufo M.M., Tonacci A., Pioggia G., Raggiunti M., Ginaldi L., Gangemi S. (2021). Vitamin D deficiency, osteoporosis and effect on autoimmune diseases and hematopoiesis: A review. Int. J. Mol. Sci..

[12] Mauricio D., Alonso N. (2024). Chronic Complications of Diabetes Mellitus: Current Outlook and Novel Pathophysiological Insights.

[13] Wang L., Zhang D., Xu J. (2020). Association between the Geriatric Nutritional Risk Index, bone mineral density and osteoporosis in type 2 diabetes patients. J. Diabetes Investig..

[14] Kanis J.A. (1994). Assessment of fracture risk and its application to screening for postmenopausal osteoporosis: Synopsis of a WHO report. WHO Study Group. Osteoporos. Int..

[15] Bouillanne O., Morineau G., Dupont C., Coulombel I., Vincent J.P., Nicolis I., Benazeth S., Cynober L., Aussel C. (2005). Geriatricnutritional risk index: A new index for evaluating at-risk elderly medical patients. Am. J. Clin. Nutr..

[16] Muraki S., Yamamoto S., Ishibashi H., Oka H., Yoshimura N., Kawaguchi H., Nakamura K. (2007). Diet and lifestyle associated with increased bone mineral density: Cross-sectional study of Japanese elderly women at an osteoporosis outpatient clinic. J Orthop. Sci..

[17] Zhao Y., Chen C., Lv X., Li K., Wang Y., Ma D., Zan X., Han M., Guo X., Fu S. (2024). Association of geriatric nutritional risk index with bone mineral density and osteoporosis in postmenopausal elderly women with T2DM. Asia Pac. J. Clin. Nutr..

[18] Li H., Xu J., Zhao Z. (2022). Association between GNRI and osteoporosis in older adults: A systematic review and meta-analysis. Clin. Nutr..

[19] Ren M., Sheng Q., Xie X., Zhang X., Han F., Chen J. (2020). Geriatric nutritional risk index is associated with mortality in peritoneal dialysis patients. Intern. Med. J..

[20] Wang T., Wang Y., Liu Q., Guo W., Zhang H., Dong L., Sun J. (2024). Association Between Geriatric Nutrition Risk Index and 90-Day Mortality in Older Adults with Chronic Obstructive Pulmonary Disease: A Retrospective Cohort Study. Int. J. Chron. Obstruct. Pulmon. Dis..

[21] Shu A., Yin M.T., Stein E., Cremers S., Dworakowski E., Ives R., Rubin M.R. (2012). Bone structure and quality in type 2 diabetes mellitus. J. Bone Min. Res..

[22] Napoli N., Chandran M., Pierroz D.D., Abrahamsen B., Schwartz A.V., Ferrari S.L., IOF Bone and Diabetes Working Group (2017). Mechanisms of diabetes-induced bone fragility. Nat. Rev. Endocrinol..

[23] Vestergaard P. (2008). Discrepancies in bone mineral density and fracture risk in diabetes. BMJ.

[24] Nielsen B.R., Abdulla J., Andersen H.E., Schwarz P., Suetta C. (2018). Sarcopenia and osteoporosis in older people: A systematic review and meta-analysis. Eur. Geriatr. Med..

[25] Wong R.M.Y., Wong H., Zhang N., Chow S.K.H., Chau W.W., Wang J., Chim Y.N., Leung K.S., Cheung W.H. (2019). The relationship between sarcopenia and fragility fracture—A systematic review. Osteoporos. Int..

[26] Starup-Linde J. (2015). Diabetes, bone, and glucose-lowering agents: A clinical update. Diabetologia.

[27] Kanis J.A., Cooper C., Rizzoli R., Reginster J.Y., Scientific Advisory Board of the European Society for Clinical and Economic Aspects of Osteoporosis (ESCEO), Committees of Scientific Advisors and National Societies of the International Osteoporosis Foundation (IOF) (2019). European guidance for the diagnosis and management of osteoporosis. Osteoporos. Int..

[28] Bonjour J.P. (2011). Protein intake and bone health. Int. J. Vitam. Nutr. Res..

[29] Ginaldi L., De Martinis M. (2015). Inflammaging and bone loss. Immun. Ageing.

